# Component characterization of *Smilax glabra* Roxb., and its inhibitory activity against *Helicobacter pylori* through targeted suppression of its secreted urease

**DOI:** 10.3389/fcimb.2025.1617330

**Published:** 2025-07-25

**Authors:** Ying Tang, Fan Yang, Xia Wen, Yi Zhou, Rong Tang, Xiuzhi He, Qiang Lu, Cailan Li

**Affiliations:** ^1^ Department of Pharmacology, Zunyi Medical University, Zhuhai, China; ^2^ Department of Pharmaceutical Sciences, Zunyi Medical University, Zhuhai, China; ^3^ Key Laboratory of Basic Pharmacology of Ministry of Education and Joint International Research Laboratory of Ethnomedicine of Ministry of Education, Zunyi Medical University, Zunyi, China; ^4^ Key Laboratory of Basic Pharmacology of Guizhou Province and School of Pharmacy, Zunyi Medical University, Zunyi, China

**Keywords:** *Smilax glabra* Roxb., astilbin, *Helicobacter pylori*, urease, thiol, molecular docking

## Abstract

**Background:**

*Smilax glabra* Roxb. (SGR), known as “tufuling” in China, is a medical and edible plant, which has anti-inflammatory, antibacterial and antineoplastic activity. SGR is extensively utilized in the remedy of gastroenteric disorders associated with *H. pylori* infection. However, the precise mechanism underlying the anti-*H. pylori* function of SGR remains to be elucidated.

**Aim:**

The inhibitory impact of SGR on the growth of *H. pylori* was examined. Subsequently, SGR against *H. pylori* urease (HPU) and jack bean urease (JBU) was investigated to illuminate the inhibitory effects, kinetic types, sites of inhibition, and potential mechanisms of action.

**Methods:**

UPLC-ESI-MS/MS was applied to identify the components of SGR. The anti-*H. pylori* effect of SGR was conducted by agar dilution method. The enzyme inhibitory activities of SGR and its primary constituents were assessed through a modified spectrophotometric Berthelot (phenol-hypochlorite) assay. The kinetics of urease inhibition were analyzed using Lineweaver-Burk plots. To explore the underlying mechanisms, sulfhydryl group reagents and Ni^2+^ binding depressors were employed. Additionally, molecular docking simulations were conducted to examine the binding interactions between the main compounds of SGR and urease.

**Results:**

A total of 34 compounds including astilbin, engeletin, isoengeletin, neoastilbin, isoastilbin and neoisoastilbin are identified in SGR. SGR was observed to inhibit the growth of three *H. pylori* strains (ATCC 43504, NCTC 26695, and ICDC 111001) with minimum inhibitory concentration (MIC) values spanning a range of 0.5 to 1.5 mg/mL. Moreover, SGR exerted a significant inhibitory effect on HPU and JBU, with IC_50_ values of 1.04 ± 0.01 mg/mL and 1.01 ± 0.01 mg/mL, separately. Enzyme kinetics analysis showed that SGR was a slow binding, non-competitive depressor to HPU, and a slow binding, mixed depressor to JBU. In-depth mechanism exploration showed that thiol compounds had better protective effect on HPU or JBU than inorganic substances, implying that the active site of SGR repressing urease may be the sulfhydryl group. Furthermore, glutathione reactivated SGR-inhibited urease, demonstrating that the inhibition was reversible. Additionally, astilbin and engeletin exhibited a certain inhibitory role towards urease activity, with astilbin inhibiting urease more than three times as strongly as engelitin. Enzyme kinetics analysis established that the inhibitory role of astilbin on enzymes was consistent with that of SGR. Molecular docking study indicated that astilbin and engeletin interacts with sulfhydryl groups at the active site of urease.

**Conclusion:**

These results indicated that SGR could prominently inhibit *H. pylori* growth through targeted suppression of its secreted urease. This investigation provides substantial experimental evidence supporting the consideration of SGR as a safe and promising natural treatment for *H. pylori*-associated gastrointestinal diseases.

## 1 Introduction

*Helicobacter pylori* (*H. pylori*) is a kind of gram-negative, spiral, and microaerobic bacteria. Epidemiological studies showed that nearly 50% of the world’s population is infected with *H. pylori* (Tshibangu-Kabamba and Yamaoka, 2021). Numerous studies have demonstrated that *H. pylori* is an important pathogenic factor in both acute and chronic gastritis, as well as peptic ulcers (Koch et al., 2023; Taillieu et al., 2023). Furthermore, *H. pylori* is tightly relevant to the development of gastric carcinoma and gastric lymphoma, which leads to its classification as a class ι carcinogen by WHO (de Martel et al., 2020; Feng et al., 2023).

Urease (EC 3.5.1.5), a nickel-reliant metalloenzyme, is predominantly found in bacteria, fungi, microorganisms, and diverse plants and soils (Kurdi and M-Ridha, 2023). The key to the urease activity lies in its active center, which contains two nickel ions (Ni²^+^) coordinated with carboxylated lysine and bound to a flexible fragment flap region within the molecular structure of the urease (Kappaun et al., 2018; Zambelli et al., 2011). The presence of Ni^2+^ in the active center and sulfhydryl groups are crucial for urease catalytic capacity (Cunha et al., 2021; Mazzei et al., 2021b). Additionally, urease possesses the capability to hydrolyze and generate substantial quantities of ammonia, which has adverse effects in various fields, such as medicine, agriculture, and animal husbandry (Duff et al., 2022; Ryvchin et al., 2021). Particularly, in the medical field, the urease generated by *H. pylori* catalyzes the breakdown of urea through a series of reactions, producing a significant amounts of carbon dioxide and NH_3_, which in turn promotes the development and progression of inflammation (Naz et al., 2020; Yang et al., 2022). Besides, excess of ammonia has the potential to neutralize stomach acid and promote *H. pylori* growth, leading to gastritis, ulcers, lymphoma, and other *H. pylori-*related diseases (Guo et al., 2020). Moreover, research has indicated that ureolytic bacteria that secrete urease are closely associated with urinary tract conditions, including kidney and bladder stones (Svane et al., 2024; Wagenlehner et al., 2020). Inhibition of urease activity has been established as an effective approach for preventing and treating gastrointestinal diseases and urinary tract infections (Heylen et al., 2024; Kanlaya and Thongboonkerd, 2022). Therefore, the search for therapeutic *H. pylori* infection-associated drugs through the repression of *H. pylori* urease (HPU) activity is a major focus of current researchers.

*Smilax glabra* Roxb. (SGR), known as “tufuling” in China, is a common plant from Liliaceae in China (Zhao et al., 2020). SGR has important edible values, and its rhizome is often used to stew nutritious soup, soak wine, and make guiling jelly. Moreover, the dried rhizome of SGR was a common Chinese herbal medicine which possesses many effects such as detoxification, dehumidification and joint relief (Fayad et al., 2021; Wang et al., 2017). Modern pharmacological researches have demonstrated that SGR mainly possessed immune regulation (Guo et al., 2024b), anti-inflammatory (Huang et al., 2023), anti-oxidant (Zhao et al., 2020), anti-bacterial (McMurray et al., 2020), anti-gastric cancer (Guo et al., 2024a) and analgesic properties (Ilyas et al., 2024). Clinically, it is extensively utilized in the treatment of chronic gastritis and musculoskeletal pain (Bao et al., 2018; Wu et al., 2022). Furthermore, flavonoids and flavonoid glycosides, including astilbin, neoastilbin and engeletin, were the main active ingredients, contributing to the anti-bacterial, anti-inflammatory, and analgesic activities of SGR (Lu et al., 2015; Shi et al., 2023). Additionally, Wang et al. found SGR to be a significant depressor of *H. pylori* during preliminary screening of traditional Chinese herbal remedies (Wang et al., 1994).

Thus, numerous researchers have established that SGR is beneficial in the remedy of gastrointestinal disorders. However, the pharmacological effects and mechanisms of SGR and its ingredients against HPU have not been clarified. Therefore, this study aimed to probe the repression and underlying mechanism of SGR extract against HPU through enzyme activity assay, kinetic experiment, inhibition site investigation, and molecular docking. This study will help to elucidate the effective substances and mechanism of SGR against *H. pylori*, and will provide a vital foundation for the exploitation of innovative anti-*H. pylori* drugs and novel urease depressors from traditional Chinese medicine.

## 2 Materials and methods

### 2.1 Chemicals and reagents

Campylobacter agar medium was purchased from Thermo Fisher Scientific. Acetylhydroxamic acid (AHA), jack bean urease (JBU, type III with specific activity 40.3 U/mg solid) and urea were obtained from Sigma Aldrich. Boric acid (BA) and sodium fluoride (NaF) were purchased from Maclin (Shanghai, China). Dithiothreitol (DTT) and L-cysteine (L-cys) were obtained from Solaibao (Beijing, China). Glutathione (GSH) was obtained from Meilun (Dalian, China). HEPES (Amresco >99%) was from BioFroxx. All chemicals and reagents were of analytic purity.

### 2.2 Preparation of herbal extract

SGR was purchased from Zunyi (Guizhou, China) and authenticated by one of our authors (Qiang Lu). A voucher specimen has been deposited at the Zhuhai Campus of Zunyi Medical University for reference (No. 20240516). The materials were crushed using swing grinder. Medicinal powder was extracted with 70% ethanol in 1:15 (g: mL) ratio using hot reflux method, which was followed by successive repeated twice. The extracting solution was filtrated via a 200-mesh sieve and centrifuged at 8000 rpm for 20 minutes. The resulting supernatant was then concentrated and lyophilized under vacuum conditions. Additionally, dried sample in a loose or powdered state is viewed as the standard and stored at -4 °C.

### 2.3 UPLC-MS/MS analysis

SGR extraction was dissolved in acetonitrile and filtrated through 0.22 μm microporous membrane. The Acquity UPLC system equipped with the Waters Xevo G2 Q-Tof system integrated with a switchable electrospray ion source interface (ESI) was used to UPLC-MS/MS analysis. Analytical separation was conducted by Acquity UPLC BEH C18 (100 mm×2.1 mm, 1.7 μm). The mobile phase is acetonitrile (eluent A) and formic acid aqueous solution (eluent B), with a linear gradient elution: 0∼2.0 min, 3%A;2.0∼12.0 min, 3%~21%A; 12.0∼17.0 min, 21%~46%A; 17.0∼25.0 min, 46%~70%A; 25.0∼28.0 min, 70%~100%A, at a flowrate of 0.4 mL/min. The injecting volume was 5 μL and the column oven temperature was 40°C. Complete ESI positive ionization scanning from m/z 50-1200Da. Data collection and analysis were performed using TOF-MS^e^ software and Peak View 1.2 software, and the main active components of SGR were deduced according to the precise molecular weight and secondary fragment information.

### 2.4 *H. pylori* strains and preparation of HPU

*H. pylori* was inoculated in Campylobacter agar and grown on Columbia agar with appropriate bovine serum albumin at 37°C, 98% humidity, and low aerobic conditions (5% O_2_, 10% CO_2_, and 85% N_2_) for 72 hours. Three days later, *H. pylori* was collected by scraping and then suspended in phosphate buffered saline (PBS). Besides, the *H. pylori* concentration was calibrated to 1×10^8^ CFU/mL by turbidimetric method. Standard HPU was extracted from *H. pylori* strain ATCC 43504 following the approach detailed by Matsubara et al. (2003). The resulting HPU preparation represents a crude enzyme extract, which has been widely adopted for initial inhibitor screening studies (Li et al., 2018; Tan et al., 2017; Yu et al., 2015). All inhibition assays included appropriate controls (enzyme blanks and solvent controls) to ensure specific detection of urease activity.

### 2.5 Minimal inhibitory concentration assay

In this study, three *H. pylori* strains—ATCC 43504, NCTC 26695, and ICDC 111001—were utilized to evaluate the antimicrobial activity of SGR. Mueller-Hinton blood agar plates were prepared with varying SGR concentrations ranging from 0 to 1.5 mg/mL. A 100 μL suspension of each *H. pylori* strain was inoculated onto the respective SGR-supplemented plates. Positive controls (metronidazole) and negative controls (solvent water) were included in experiments. The plates were then incubated under microaerophilic conditions for three days. The minimum inhibitory concentration (MIC) was defined as the lowest concentration of SGR or metronidazole at which no bacterial growth was observed compared to negative control wells.

### 2.6 Standard urease activity test

The protein concentration of HPU was tested utilizing BCA protein detection kit. Standard urease test mixture consists of 150 mM urea in HEPES buffer (20 mM). HPU solution of varying concentrations was mixed with 150 mM urea as the substrate in a HEPES buffer (20 mM), and then reacted at 37 °C for 20 min. Urease vitality was assessed based on ammonia levels generated during the reaction. Ultimately, residual urease activity was measured by modified Berthelot (phenol hypochlorite) at 595 nm. This experiment was performed three times in parallel. The result revealed that the HPU activity was determined to be 17.0 U/mg compared to JBU (40.3 U/mg).

### 2.7 Inhibition experiment of urease activity

Test drug solution with equal volume and different concentration was mixed with urease and incubated in a 96-well plate at 37°C for 20 min. AHA was used as the positive control, while urease with urea (no depressor) served as the negative control. Moreover, urea solution (150 mM) was admixed and coincubated at ambient temperature for 20 min. Residual urease activity was measured via using the modified Berthelot method. The percentage of residual activity (RA%) was calculated as (A^sample^ - A^blank^)/(A^negative control^ - A^blank^)×100%, where A^blank^ represents the background absorbance without urease. Calculating the half-maximal inhibitory concentration (IC_50_) of the depressor to assess the impact of the test drug towards enzyme activity. Each experiment was conducted in triplicate for validation.

### 2.8 Determination of inhibition type

Residual urease activity was determined by pre-incubating test drug, urease mixture with a series of urea concentrations. Michaelis constant (*K_M_*) and maximum velocity (*v_max_*) values are obtained from the Lineweaver-Burk plots of 1/v and 1/urea by plotting the reciprocal reaction velocity and substrate concentration. Variation characteristics of urease kinetic parameters *K_M_* and *v_max_* were analyzed by adding different concentrations of test products to determine the type of inhibiting effect on HPU. Each sample was performed in triplicate.

### 2.9 Analysis of reaction progress curve

A functional relationship was established by measuring the impact of incubation time on ammonia concentration in the presence or absence of test drug solution. Before the reaction began, test drug mixed with urease was immediately reacted in a non-pre-incubated system. In contrast, test product was mixed with urease and incubated for 20 minutes prior to the addition of urea to the pre-incubation system. Urease vitality was determined according to standard measurements at various time points. Using a curve-fitting computer program, the experimental points are fitted into the following integral equation describing the progression curve:


$$\text{P}\left(\text{t}\right)={\text{V}}_{\text{s}}\text{t}+\left({\text{V}}_{0}-{\text{V}}_{\text{s}}\right)\left(1-{\text{e}}^{-{\text{k}}_{\text{app}}\text{t}}\right)/{\text{K}}_{\text{app}}$$

Where, P_t_ represents the accumulated product yield at time t and 0, V_0_ and V_s_ denote the initial and steady-state velocities of the reaction, while k_app_ stands for the apparent velocity constant.

### 2.10 Protective assay of the SGR-inhibiting enzyme

#### 2.10.1 Impact of thiol compounds on SGR inhibition of urease

Urease was combined with SGR solution (1.5 mg/mL), incubated for 20 minutes, and then sulfhydryl compounds (DTT, GSH and L-cys) were added. Experiment was repeated three times simultaneously.

#### 2.10.2 Impact of inorganic compounds on SGR inhibition of urease

After incubating mixture of urease and SGR solution (1.5 mg/mL) for 20 min, inorganic compounds including 1.25 mM BA or NaF were added. Experiment was repeated three times simultaneously.

### 2.11 SGR-thiol-urease interplay assay

#### 2.11.1 Effect of incubating time towards urease vitality

The mixture containing urease, SGR solution (1.5 mg/mL), and 1.25 mM sulfhydryl compound (DTT, GSH and L-cys) was coincubated at 37 °C in 20 mM HEPES buffer for 5, 10, 20, and 40 min, respectively, removed at each time point, and measured residual urease activity. Assay was repeated three times simultaneously.

#### 2.11.2 Impact of adding order towards urease activity

Culture mixture consists of HPU, and 1.25 mM sulfhydryl compound (DTT, GSH, or L-cys) in 20 mM HEPES buffer. Each test was carried out in parallel three times.

The ingredients of the culture mixture are blended as follows:

1. SGR solution and sulfhydryl compound were co-incubated for 20 min, followed by the introduction of urease.
2. Urease was incubated with sulfhydryl compound for 20 min and then added into SGR solution.
3. Urease was incubated with SGR solution for 20 min, followed by the introduction of sulfhydryl compounds.

### 2.12 Reactivation of depressor-inactivated urease

Urease in the mixture was pre-incubated with the SGR solution (2 mg/mL) for 20 minutes. Subsequently, 1.25 mM GSH was introduced and coincubated with the pre-incubator to detect residual urease activity of the mixture at diverse time intervals. Residual enzyme vitality was inspected both before and after the introduction of GSH. Experiment was carried out three times in parallel.

### 2.13 Molecular docking analysis

Molecular docking software AutoDock Vina 1.1.2 was utilized to analyze the potential binding sites of astilbin and engeletin to urease. The selection of these two ureases was based on their complementary biological relevance: HPU (PDB ID: 1E9Y, resolution: 3.00 Å) represents the primary therapeutic target for *H. pylori* infection, while JBU (PDB ID: 3LA4, resolution: 2.05 Å) serves as a well-characterized reference with conserved catalytic domains that facilitates comparative mechanistic analysis. The 3D structure of astilbin and engeletin was obtained from PubChem database. The compound and target protein formats were converted to PDBQT files using AutoDockTools 1.5.6 software. Before docking, all water molecules were eliminated, and hydrogen atoms were placed on the receptors and given an electric charge. A cubic grid box of 60 × 60 × 60 Å with 0.375 Å spacing was centered at the average coordinates of the two Ni²^+^ ions (for HPU X=127.864, Y=126.349, Z=87.546; for JBU X=-39.959, Y=-44.679, Z=-74.986) to cover the entire active site. Docking employed the Lamarckian GA (10 runs, exhaustiveness=8) with Vina’s scoring function. Method validation confirmed reproducibility (RMSD< 2.0 Å for re-docked ligands). The resulting binding poses were analyzed using PyMOL for 3D visualizations, with binding energies compared between targets to elucidate species-specific interactions.

### 2.14 Statistical analysis

In this study, GraphPad Prism 13.0 software was utilized for data visualization. Data are presented as mean ± standard error (S.E.M). SPSS 29.0 software was employed for statistical analysis. Results were processed applying one-way analysis of variance (ANOVA) to determine statistical differences between groups, followed by the Dunnett test. The significance degree was set at *p*< 0.05.

## 3 Results

### 3.1 UPLC-ESI-MS/MS analysis

**Figure 1:**
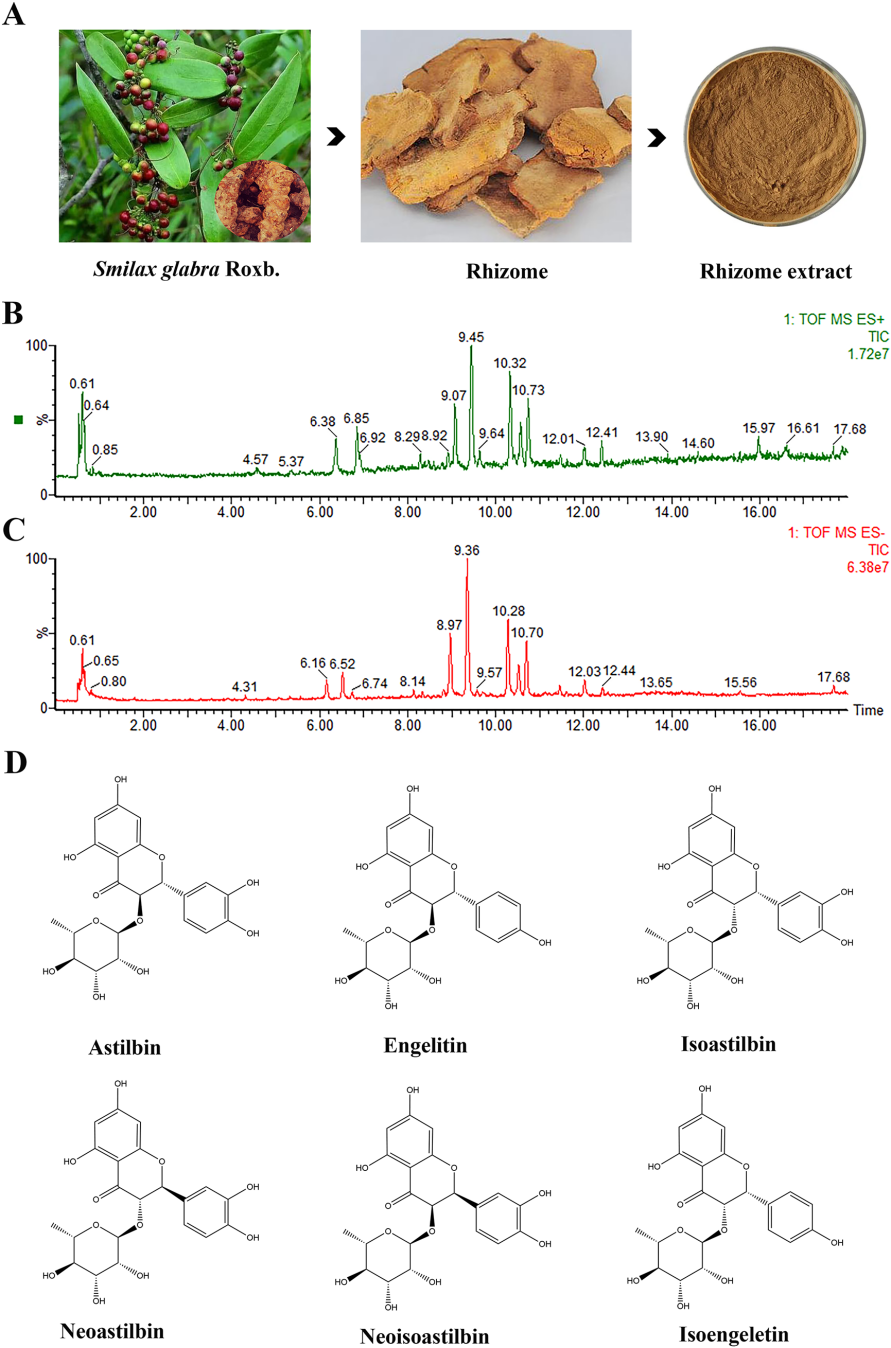
Chemical composition analysis of *Smilax glabra* Roxb. (SGR). **(A)** The plants, rhizomes, and 70% ethanol extracts of SGR. Total ion chromatogram of SGR in positive **(B)** and negative ion mode **(C)**. **(D)** Flavonoids including astilbin, engeletin, isoengeletin, neoastilbin, isoastilbin, neoisoastilbin was identified as the main chemical component of SGR.

As illustrated in **Figure 1** and **Table 1**, UPLC-ESI-MS/MS test revealed the 40 compounds including astilbin, engeletin, isoengeletin, neoastilbin, isoastilbin, and neoisoastilbin were identified in the SGR extract under positive and negative ion mode analysis. Moreover, the compounds can be roughly classified as organic acids, flavonoids, phenols, sesquiterpenes, etc., according to the precise molecular weight and secondary fragment information.

**Table 1:** Peak assignment of SGR extracts using LC-MS/MS in positive and negative ionization modes.

No.	Component name	Formula	Adducts	t_R_ (min)	Calculated (m/z)	MS/MS	Classify
1	Shikimic acid	C_7_H_10_O_5_	-H	0.66	173.0456	137.0247, 78.9603	Organic acid
2	Malic acid	C_4_H_6_O_5_	-H	0.67	133.0143	72.9946	Organic acid
3	Epicatechin	C_15_H_14_O_6_	-H	4.33	289.0718	271.0636, 245.0812, 179.0351, 137.0242, 109.0298	Flavonoids
4	Procyanidin B1	C_30_H_26_O_12_	-H	5.59	577.1352	425.0882, 407.0762, 289.0717, 161.0249	Flavonoids
5	5-O-Caffeoylshikimic acid	C_16_H_16_O_8_	-H	5.97	335.0772	179.0359, 137.0253, 135.0454, 93.0358	Phenols
6	8-O-Caffeoylshikimic acid	C_16_H_16_O_8_	-H	6.14	335.0772	289.0719, 245.0816, 203.0711	Phenols
7	Catechin	C_15_H_14_O_6_	-H	6.16	289.0718	161.0248, 137.0248, 109.0301	Flavonoids
8	1,8-Dihydrxy-3,5-dimethoxyxanthone	C_15_H_12_O_6_	-H	6.17	287.0562	137.0248, 109.0301	Flavonoids
9	4-O-Caffeoylshikimic acid	C_16_H_16_O_8_	-H	6.53	335.0772	179.0356, 161.0247, 135.0456	Phenols
10	Smiglanin	C_15_H_16_O_9_	-H	7.44	339.0722	192.0066, 136.0168, 80.0278	Flavonoids
11	Isomer of Astilbin	C_21_H_22_O_11_	-H	8.14	449.1089	269.0456, 151.0041, 125.0249	Flavonoids
12	Dihydrokaempferol-5-O-β-D-glucopyranoside	C_21_H_22_O_11_	-H	8.47	449.1089	269.0456, 259.0625, 151.0042	Flavonoids
13	Cinchonain Ia	C_24_H_20_O_9_	-H	8.82	451.1035	341.0668, 217.0144, 189.0198, 109.0301	Phenols
14	Isoastilbin	C_21_H_22_O_11_	-H	8.97	449.1089	303.0518, 285.0416, 151.0046, 125.0249	Flavonoids
15	Cimicifugic acid B	C_21_H_20_O_11_	-H	8.98	447.0933	303.0518, 285.0416, 151.0046	Phenylacetates
16	Isoastilbin	C_21_H_22_O_11_	+H	9.07	451.1235	343.0134, 305.0692, 153.0184, 149.0237	Flavonoids
17	Neoastilbin	C_21_H_22_O_11_	-H	9.36	449.1089	303.0525, 285.0419, 151.0048, 125.0250	Flavonoids
18	Neoastilbin	C_21_H_22_O_11_	+H	9.43	451.1235	305.0673, 153.0177, 149.0229	Flavonoids
19	Astilbin	C_21_H_22_O_11_	-H	10.28	449.1089	303.0517, 285.0417, 151.0047, 125.0249	Flavonoids
20	Astilbin	C_21_H_22_O_11_	+H	10.31	451.1235	343.0159, 195.0284, 153.0194, 149.0236	Flavonoids
21	Isomer of Cinchonain Ia	C_24_H_20_O_9_	-H	10.40	451.1035	341.0664, 289.0710, 217.0146, 189.0199	Phenols
22	Quercetin-3-O-α-L-rhamnoside	C_21_H_20_O_11_	-H	10.48	447.0933	300.0271, 271.0436, 255.0311, 145.0300	Flavonoids
23	Neoisoastilbin	C_21_H_22_O_11_	-H	10.52	449.1089	303.0509, 285.0409, 259.0612, 151.0045, 125.0248	Flavonoids
24	7-Hydroxyaloin A	C_21_H_22_O_10_	-H	10.55	433.114	341.0668, 269.0456, 178.9989, 125.0247	Benzenoid aromatic compound
25	Engeletin	C_21_H_22_O_10_	-H	10.70	433.114	287.0560, 269.0463, 259.0616,152.0120	Dihydroflavonoid glycosides
26	5,7,3’,5’-Tetrahydroxyflavanone	C_15_H_12_O_6_	+H	10.72	289.0707	153.0199,149.0235	Flavanones
27	Kaempferol-3-O-rhamnoside	C_21_H_20_O_10_	-H	11.86	431.0984	337.0894, 285.0400, 227.0367, 167.0361	Flavonol glycosides
28	Isoengelitin	C_21_H_22_O_10_	-H	12.02	433.114	287.0556, 269.0456, 259.0609, 178.9989, 152.0115	Flavonoids
29	Cinchonain Ib	C_24_H_20_O_9_	-H	12.43	451.1035	341.0663, 289.0715, 189.0194, 177.0193	Phenols
30	Isomer of Cinchonain Ia	C_24_H_20_O_9_	-H	12.68	451.1035	341.0662, 177.0192, 109.0299	Phenols
31	Germacrone	C_15_H_22_O	+H	16.00	219.1743	119.0852, 91.0539	Sesquiterpenes
32	Dehydrocurdione	C_15_H_22_O_2_	+H	20.93	235.1693	219.1402, 179.1064	Sesquiterpenes
33	Safrol	C_10_H_10_O_2_	+H	21.86	163.0754	105.0329	Hydrocarbons
34	Butyl isobutyl phthalate	C_16_H_22_O_4_	+H	22.55	279.1591	149.0228, 121.0279	Phthalic Acids

### 3.2 MIC of SGR against *H. pylori*

As illustrated in **Table 2**, SGR exhibited varying degrees of growth inhibition against the three *H. pylori* strains under a culture condition of pH 7.2. Notably, the MIC of SGR against the standard strain ATCC 43504 was determined to be 1.5 mg/mL. In contrast, the MIC values for the strains NCTC 26695 and ICDC 111001 were significantly lower, both at 0.5 mg/mL, indicating greater susceptibility of these strains to SGR’s antimicrobial effects. In comparison, the standard antibacterial agent metronidazole demonstrated significant anti-*H. pylori* activity against strain ICDC 111001, with an MIC of 2.0 μg/mL.

**Table 2:** Minimal inhibitory concentrations (MICs) of SGR against *H. pylori* strains.

Test drug	*H. pylori* strains	MIC (mg/mL)
SGR	ATCC 4304	1.5
SGR	NCTC 26695	0.5
SGR	ICDC 111001	0.5
Metronidazole	ICDC 111001	2×10^-3^

### 3.3 SGR-inhibition urease activity

**Figure 2:**
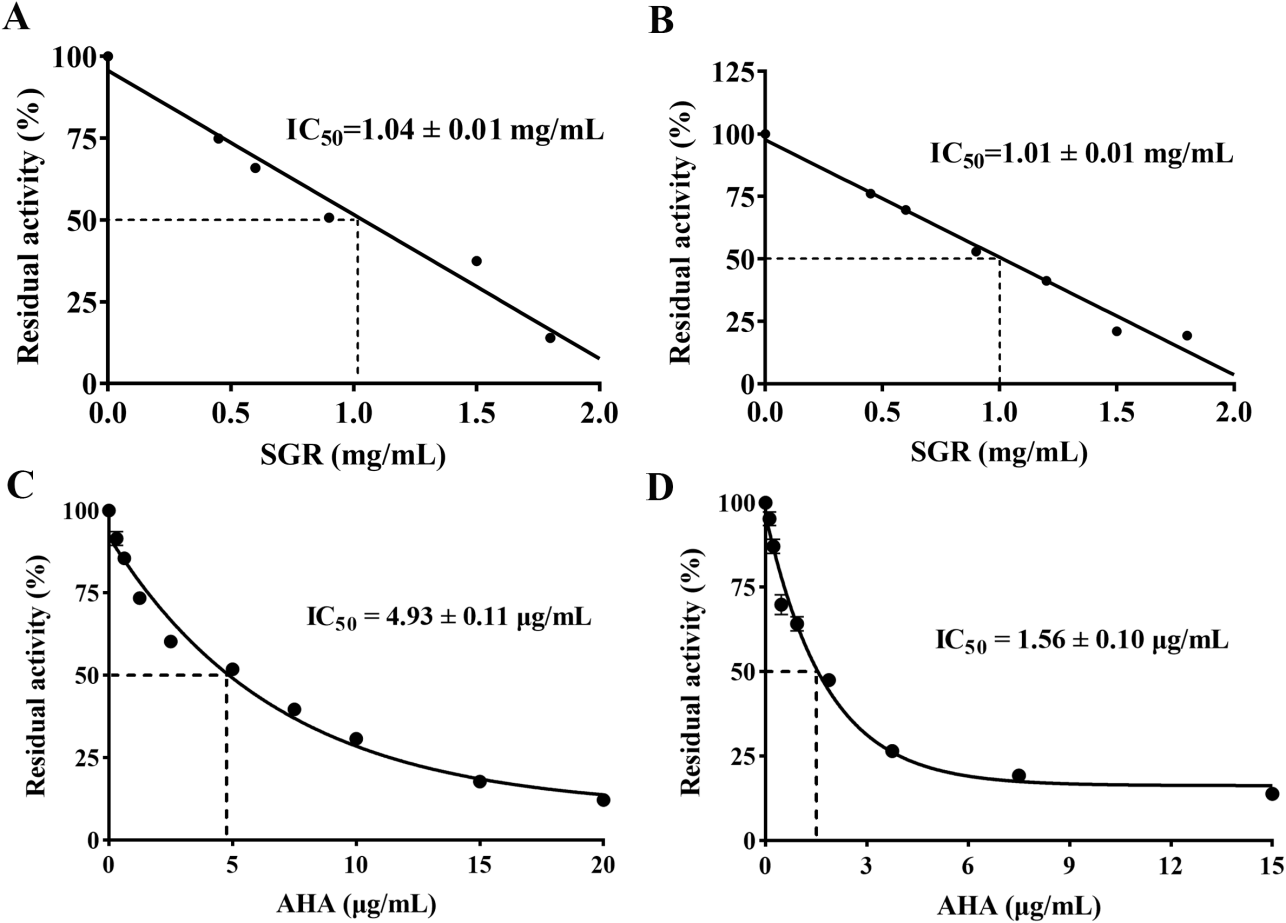
Inhibitory effects of various concentrations of SGR on HPU **(A)** and JBU **(B)**. Inhibitory role of AHA towards HPU **(C)** and JBU **(D)**. The experimental data are exhibited as means ± SEM (n = 3).

As shown in **Figure 2**, SGR demonstrated a significant inhibitory effect on HPU and JBU, with IC_50_ values of 1.04 ± 0.01 mg/mL and 1.01 ± 0.01 mg/mL, separately. In addition, the IC_50_ values of AHA, as standard urease depressor, were 4.93 ± 0.11 μg/mL and 1.56 ± 0.10 μg/mL for inhibiting HPU and JBU, separately.

### 3.4 SGR-inhibitive type analysis

**Figure 3:**
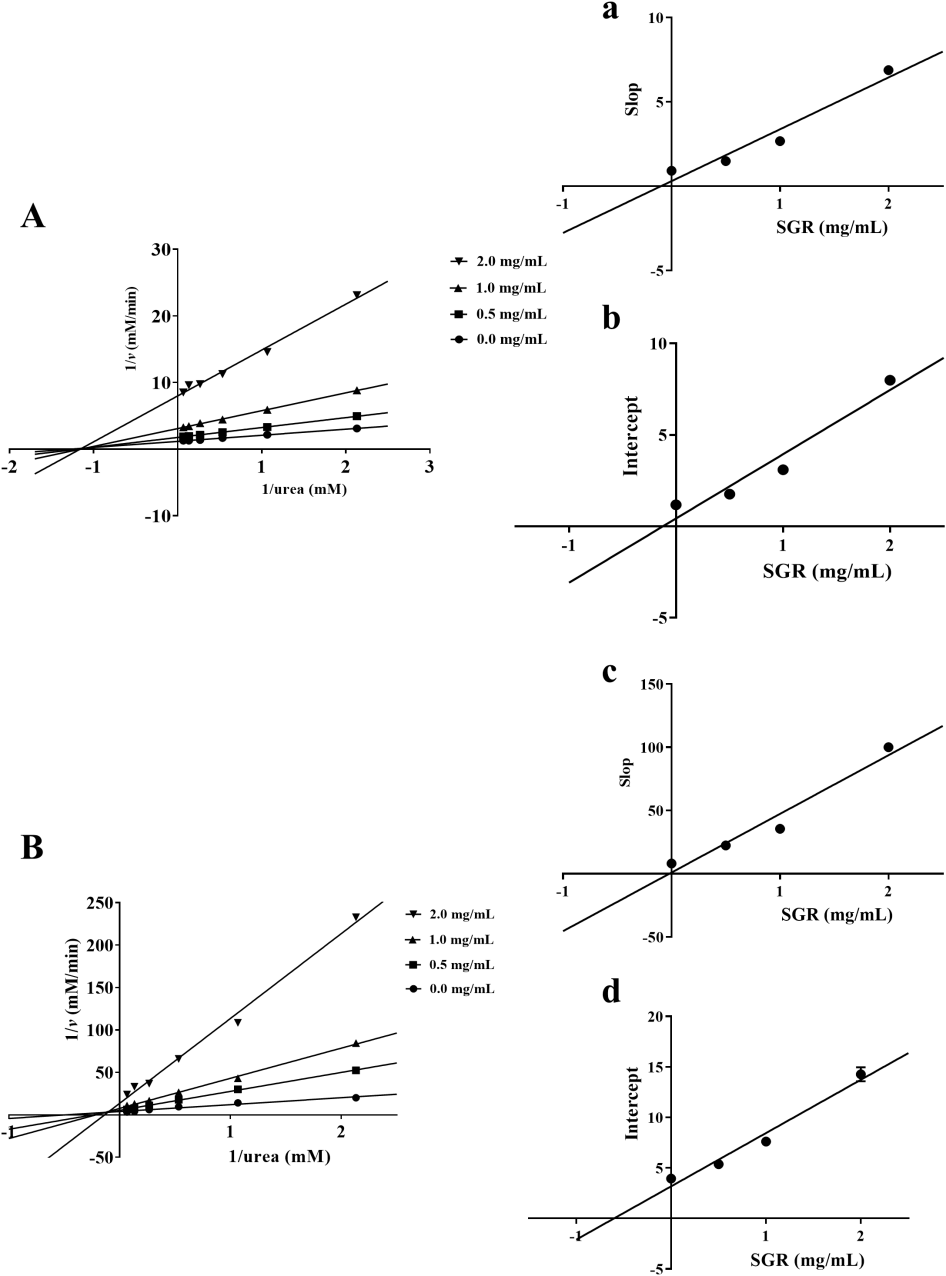
Enzymatic kinetics analysis of SGR against urease. Lineweaver-Burk plots were constructed to illustrate the inverse of reaction velocities against the reciprocal of urea concentration for HPU **(A)** and JBU **(B)**. These plots were generated in the presence of SGR at dosages of 0.0, 0.50, 1.0, and 2.0 mg/mL. **(a, c)** The inhibitive constant *K_i_* was gained by plotting the slope of the Lineweaver Burk plot against the dosages of SGR. **(b, d)** The inhibitive constant *K_is_* was gained by plotting the intercept of the Lineweaver Burk plot against the dosages of SGR. The experimental data are emerged as means ± SEM (n = 3).

As illustrated in the Lineweaver-Burk plot, all lines crossed at one location on x-axis, indicating that the kinetic parameter *K_M_* of inhibition of HPU by SGR remained basically unchanged, while the value of *V_max_* gradually decreased after adding various concentrations of SGR (**Figure 3A**). According to Lineweaver-Burk mapping analysis, the inhibitory type of SGR on HPU was non-competitive depressor. Additionally, the equilibrium parameters for binding of SGR to the free enzyme (*K_i_*) and to the enzyme-substrate complex (*K_is_*) were 0.10 ± 0.01 mg/mL and 0.12 ± 0.01 mg/mL, respectively, based on the relationship between the slope or intercept of the line in the Lineweaver-Burk diagram and the concentration of the suppressant (**Figures 3a, b**).

As depicted in **Figure 3B**, the plot of 1/v versus 1/urea consisted of multiple lines intersecting at one location in the second quadrant. *K_M_* gradually elevated and *V_max_* gradually declined following adding various concentrations of SGR. This suggested that SGR was a mixed suppressant for JBU. Moreover, the equilibrium parameters of *K_i_* and *K_is_* were 0.02 ± 0.02 mg/mL and 0.61 ± 0.08 mg/mL, separately (**Figures 3c, d**).

### 3.5 Reactive progress curves

**Figure 4:**
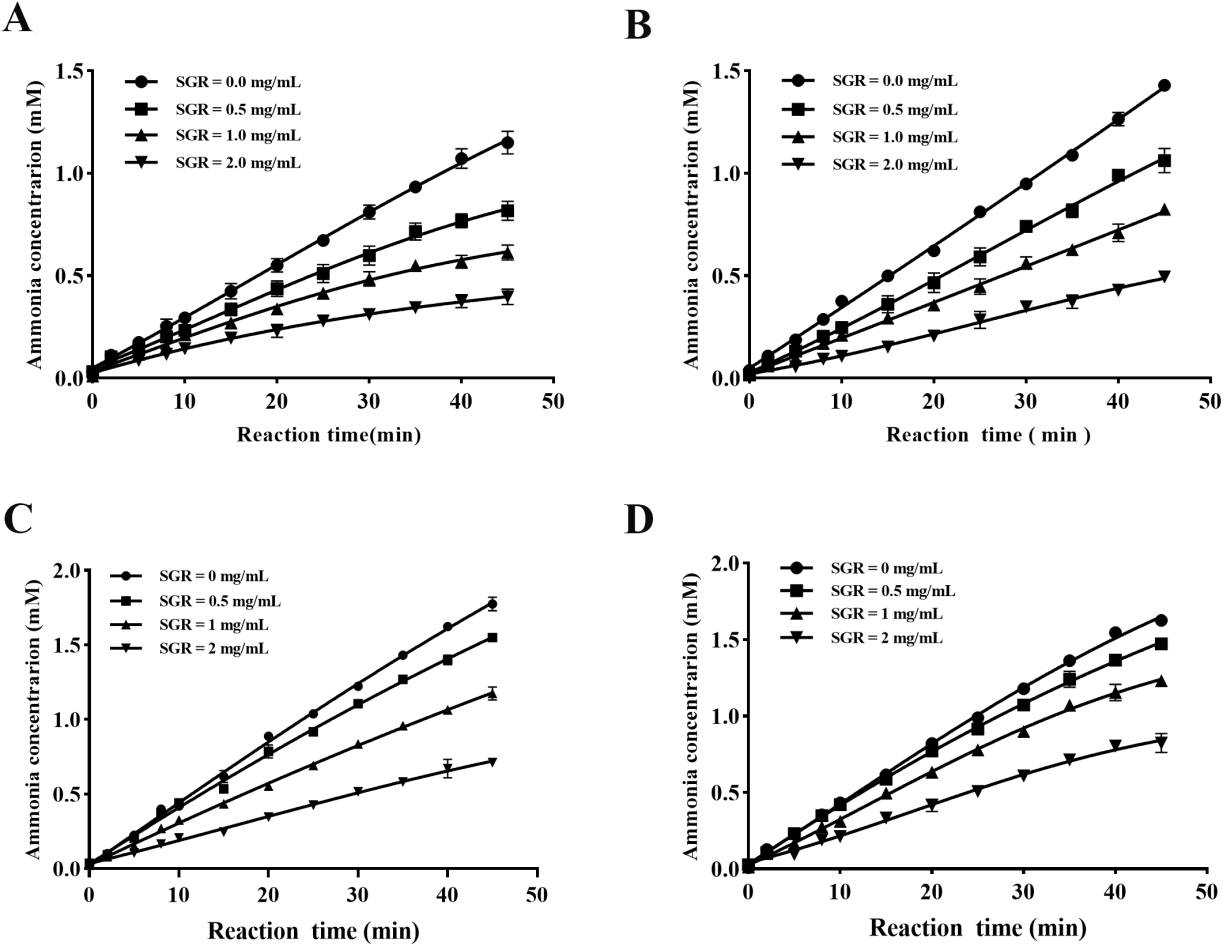
Reactive progress curves of SGR against HPU and JBU. Reaction progress curve system can be divided into non-preincubated system [HPU **(A)**, JBU **(C)**] and preincubated system [HPU **(B)**, JBU **(D)**]. Curves were generated by evaluating the correlation between ammonia amount and incubating time (0–45 minutes) in the presence of SGR at dosages of 0.0, 0.5, 1.0, and 2.0 mg/mL. Experimental data are presented as means ± SEM (n = 3).

As illustrated in **Figure 4**, depressor concentration and incubation time were found to have significant impacts towards the binding rate between SGR and urease. The curve fitting of the reaction process between SGR and HPU in unincubated and incubated system, shows a characteristic concave curve (**Figures 4A, B**), indicating rapid hydrolysis of urease at the initial velocity (V_0_). With the influence of SGR on urease, there was a gradual inhibition on urease activity, resulting in a change in the hydrolysis urease from V_0_ to steady-state velocity (V_s_) based on the first-order velocity constant (K_app_). Similarly, the reactive progress of SGR-JBU combination in unincubated and incubated system displayed a typical concave curve (**Figures 4C, D**), demonstrating that the combination had a constant equilibrium rate V_0_ from the beginning, with the hydrolysis urease rate decreasing from V_0_ to V_s_. The reactive progress curves of JBU and HPU were consistent with the slow-binding suppression depicted by Morrison and Walsh (Morrison and Walsh, 1988).

### 3.6 Protective test of the SGR-depressing enzyme

**Figure 5:**
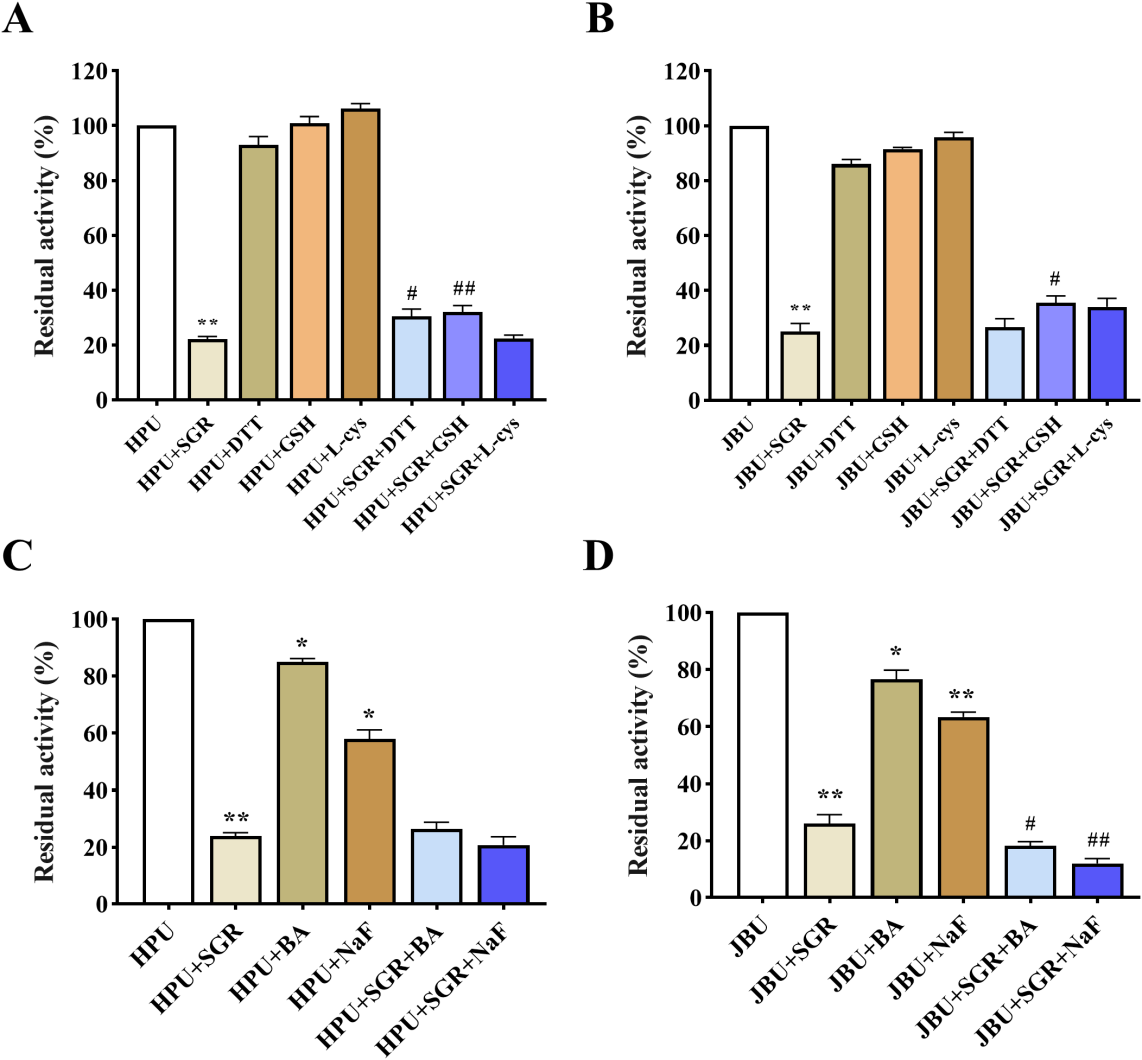
Impacts of sulfhydryl compounds on SGR-induced HPU **(A)** and JBU **(B)** inactivation. Impacts of inorganic substances on SGR-induced HPU **(C)** and JBU **(D)** inactivation. The dosages of SGR, sulfhydryl compounds (comprising DTT, GSH, and L-cys) and inorganic compounds (including NaF and BA) were 1.5 mg/mL, 1.25 mM and 1.25 mM, separately. Experimental data are emerged as means ± SEM (n = 3). *p< 0.05, **p< 0.01 *vs.* urease; #*p*< 0.05, ## *p<*0.01 *vs.* SGR group.

Three thiol-containing substances (DTT, GSH and L-cys) were utilized to probe the possible inactivation sites of SGR-induced urease. As depicted in **Figures 5A, B**, the thiol-containing compounds exhibited a higher level of activity on urease than in the free of thiol-containing substances. Therefore, the sulfhydryl group of urease may be tightly relevant to the inactivation of urease by SGR.

Numerous researches have demonstrated that the inorganic substances BA and NaF are competitive urease depressors that repress urease activity via binding to nickel ions in the active center of urease (Mazzei et al., 2019). As illustrated in **Figures 5C, D**, SGR, BA, and NaF exhibit various degrees of inhibition on urease activity. The urease activity in the SGR mixed system containing BA or NaF significantly decreased, even lower than that in the SGR group, suggesting that BA and NaF may synergistically depress the activity of HPU and JBU with SGR. Therefore, sulfhydryl compounds were shown to restore urease activity more effectively than inorganic compounds. This suggests that SGR may bind to the thiol group, which is the active site of urease.

### 3.7 SGR-thiol-urease interplay assay

**Figure 6:**
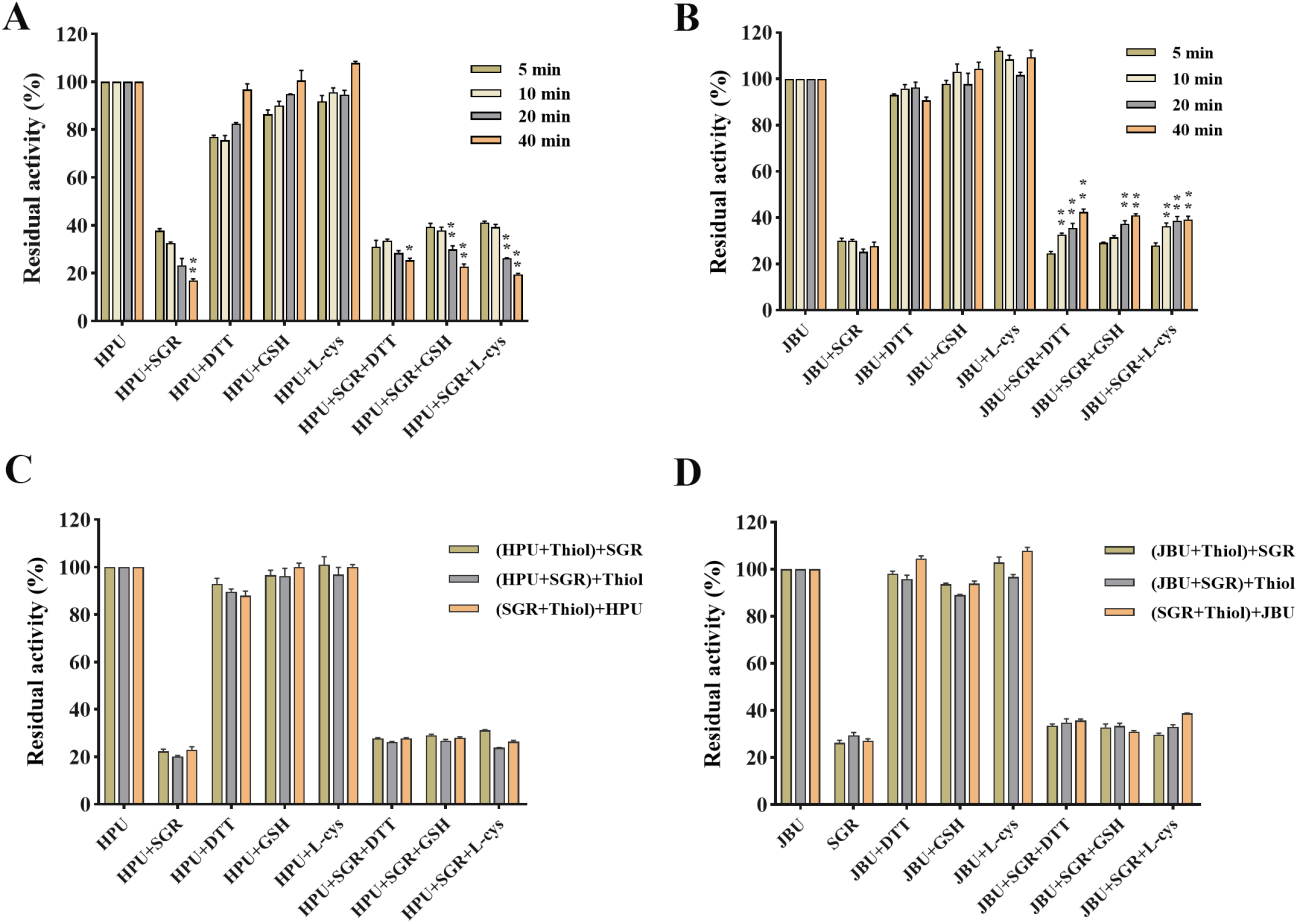
Effects of the incubation time and addition sequence of sulfhydryl reagents SGR-modified HPU **(A, C)** and JBU **(B, D)**. Enzymic activity was assessed following co-incubations for 5, 10, 20 and 40 minutes. The compound enclosed in brackets was preincubated for 20 minutes, after which the final compound (outside brackets) was introduced and incubated for another 20 minutes. The concentrations of sulfhydryl compounds and SGR were 1.25 mM and 1.5 mg/mL, separately. Data are emerged as means ± SEM (n = 3). *p< 0.05, **p< 0.01 *vs.* the first column of each group.

As seen in **Figure 6**, thiol-containing substances can alleviate the inactivation of SRG on urease. Enzyme activity was tightly relevant to the coincubation time of urease, sulfhydryl compounds, and SGR (**Figures 6A, B**). In addition, the addition sequence of urease, sulfhydryl compounds, and SGR has no significant effect on enzyme activity (**Figures 6C, D**).

### 3.8 Reactivation of SGR-inactivated urease

**Figure 7:**
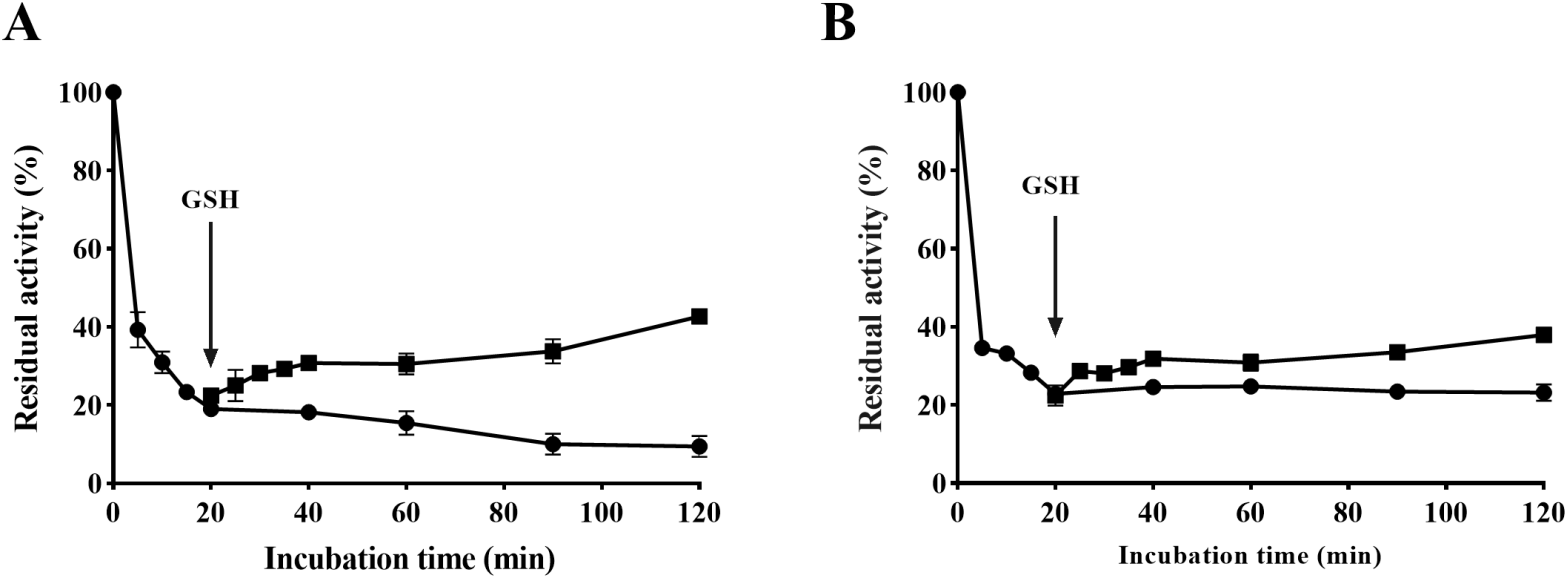
Reactivation of SGR-inactivated HPU **(A)** and JBU **(B)** with 1.25 mM GSH. The dosage of SGR repressing HPU and JBU was 1.5 mg/mL. Enzyme viability was inhibited by SGR (•) and partially recovered after GSH addition (▴).

As depicted in **Figure 7**, the urease activity decreased by approximately 80% after co-incubating urease and SGR for 20 minutes compared to its initial activity. However, with the addition of 1.25 mM GSH, HPU or JBU activities recovered approximately 40% of initial levels. The results indicated that the SGR-induced HPU or JBU reaction was reversible. The recovery of urease inhibitory activity by GSH further supports that sulfhydryl at the active site of urease exert a crucial function in the inactivation of urease by SGR.

### 3.9 Enzyme inhibitory effect of the main active ingredients of SGR

**Figure 8:**
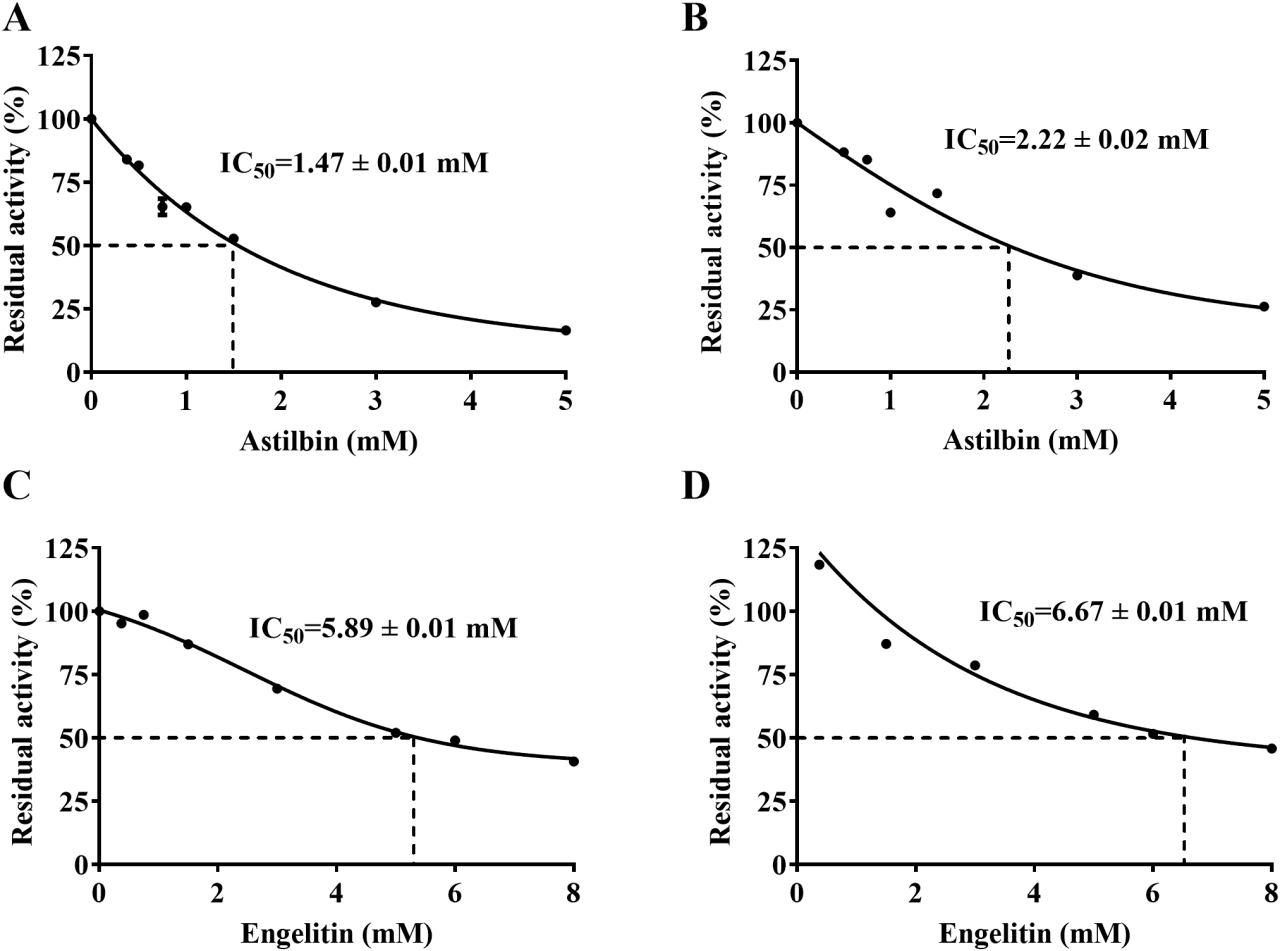
Inhibitory effect of the main active ingredients of SGR on urease. Astilbin-induced enzyme inactivation on HPU **(A)** and JBU **(B)**. Inhibitory action of engeletin towards HPU **(C)** and JBU **(D)**. Data are presented as means ± SEM (n = 3).

As depicted in **Figure 8**, the active ingredients of SGR have a good inhibitory effect on urease. The IC_50_ of astilbin depressing HPU and JBU was 1.47 ± 0.01 mM, and 2.22 ± 0.02 mM, separately. The IC_50_ of engeletin repressing HPU and JBU was separately 5.89 ± 0.01 mM and 6.67 ± 0.01 mM, suggesting that the inhibitory effect of engeletin on urease is not as effective as that of astilbin.

### 3.10 Inhibition type analysis of astilbin on urease

**Figure 9:**
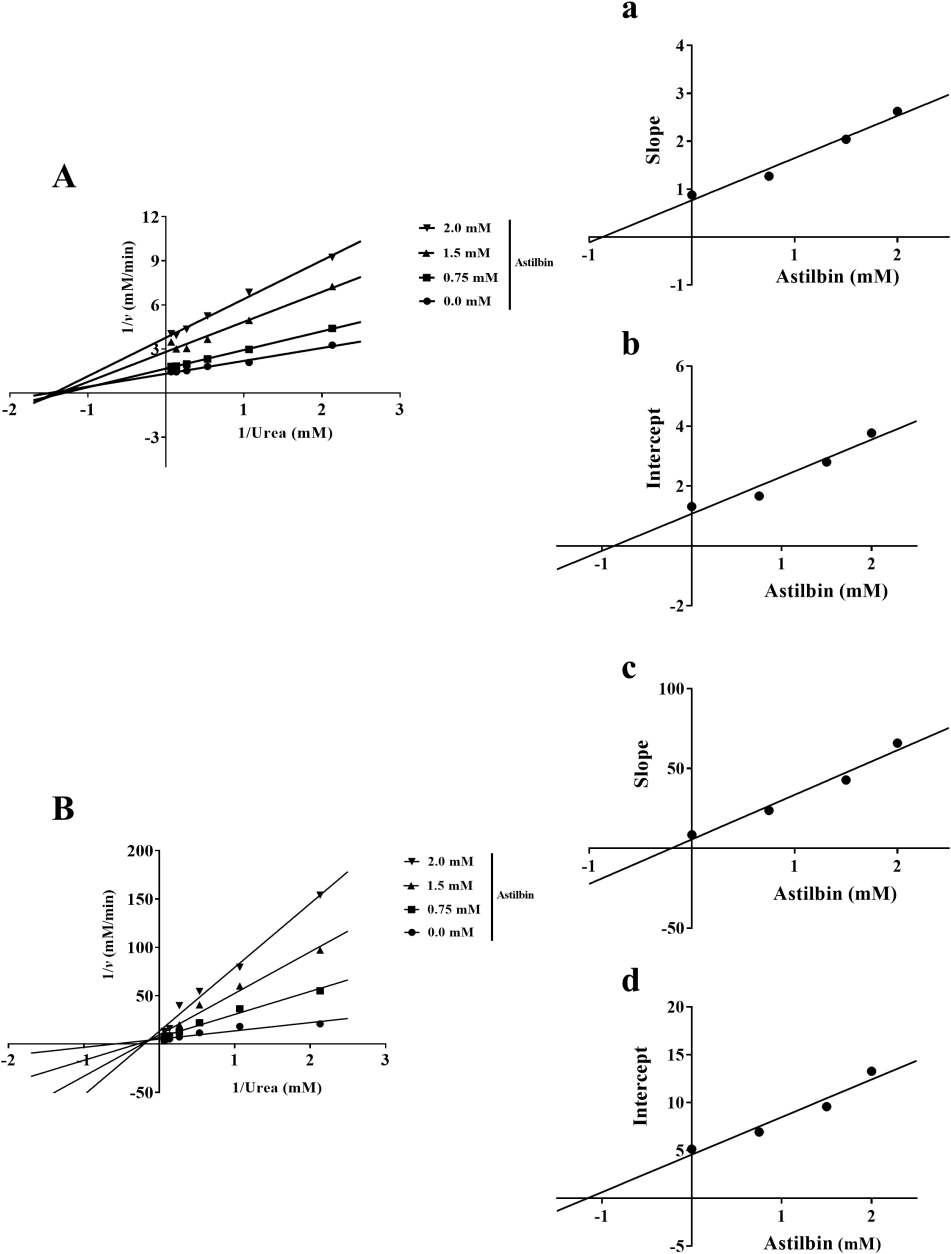
Kinetic investigation of urease inhibition by astilbin. Lineweaver-Burk plots of HPU **(A)** and JBU **(B)** were described in the non-existence and existence of diverse astilbin dosages. **(a, c)** The inhibitory parameter *Ki* was gained by plotting the slopes of Lineweaver-Burk plots versus astilbin dosages. **(b, d)** The inhibitory parameter *Kis* was gained from the plot of the intercepts of Lineweaver-Burk plots versus astilbin dosages.

As shown in **Figure 9**, the *K_M_* value did not greatly change, whereas the *v_max_* value declined with increasing astilbin concentration, implying that the inhibitory type of astilbin on HPU was non-competitive type (**Figure 9A**). The equilibrium inhibition parameters *Ki* and *Kis* were 0.87 ± 0.01 mM and 0.87 ± 0.01 mM, separately (**Figures 9a, b**). In contrast, during the binding process between astilbin and JBU, the *K_M_* value was increased while *v_max_* was decreased with increasing astilbin concentration, consistent with the kinetic characteristics of mixed inhibition (**Figure 9B**). The inhibition parameters *Ki* and *Kis* were 0.20 ± 0.01 mM and 1.17 ± 0.10 mM, separately (**Figures 9c, d**).

### 3.11 Molecular docking simulation

**Figure 10:**
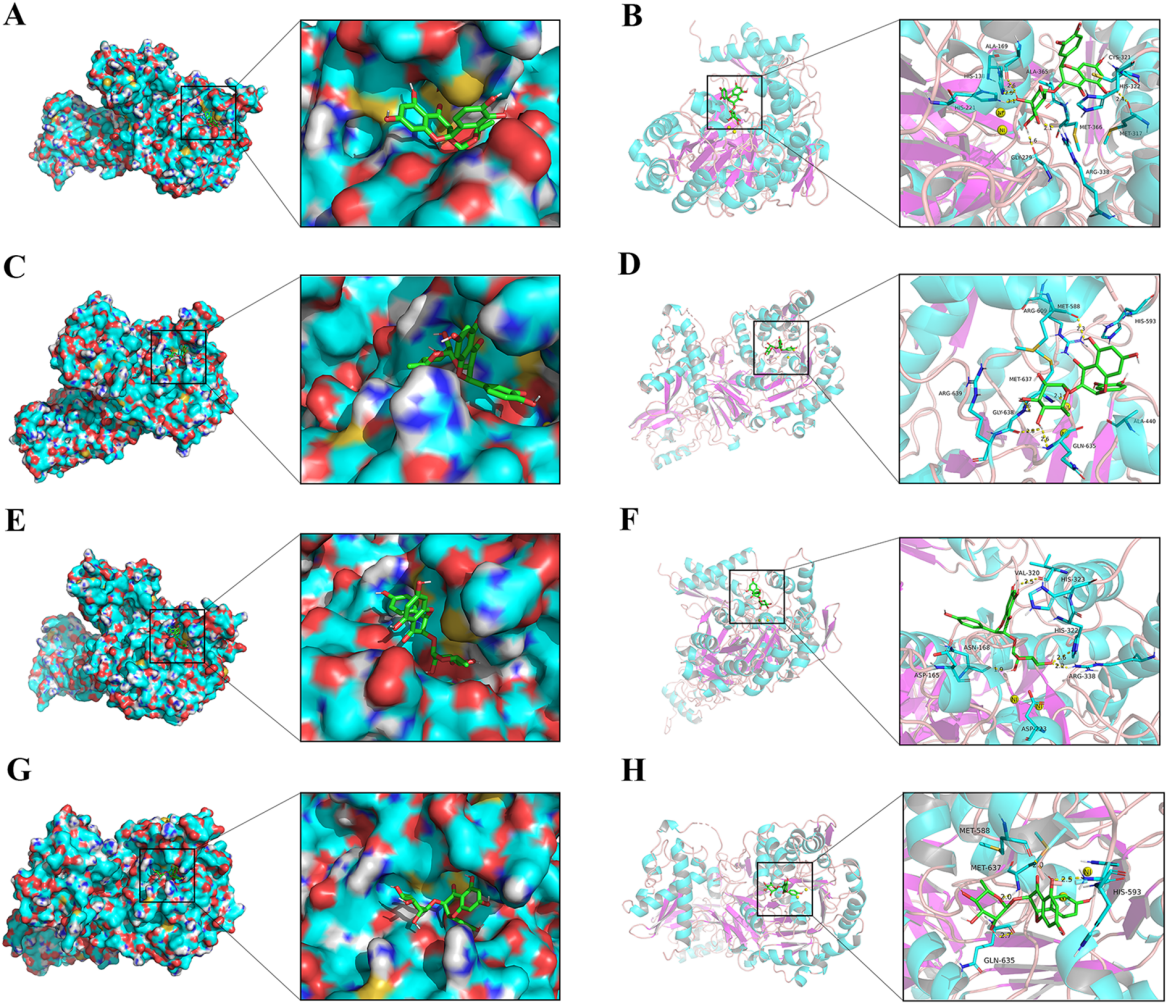
Molecular docking analysis of astilbin and engeletin with urease. Enzyme surface and cartoon mode of the interplay between astilbin and HPU **(A, B)**/JBU **(C, D)**. Enzyme surface and cartoon representations of the interaction between engeletin and HPU **(E, F)**/JBU **(G, H)**. The yellow dashed line represents hydrogen bonding interactions.

The molecular docking analysis was carried out, and the resulting interactions were visualized utilizing the Pymol software. The most probable binding modes of ligand with urease were depicted by the enzyme surface and cartoon mode. As shown in **Figures 10A-D**, astilbin exhibited docking scores of -8.0 kcal/mol for HPU and -7.6 kcal/mol for JBU. In HPU, astilbin formed hydrogen bonds with ARG 338, MET 317, HIS 138, HIS 221, GLY 279, and ALA 169 in the mobile flap region, and interacted with CYS 321, HIS 322, MET 366, and ALA 365 via hydrophobic forces, potentially stabilizing the flap in an open conformation and inhibiting catalytic activity. In JBU, astilbin hydrogen-bonded with GLY 638, MET 637, MET 588, and GLN 635, and engaged in hydrophobic interactions with HIS 593, ALA 440, ARG 609, and ARG 639, similarly stabilizing the flap in an open state and inhibiting enzyme activity.

Additionally, as illustrated in **Figures 10E–H**, engeletin demonstrated notable affinity for urease, with binding docking scores of -7.5 kcal/mol for HPU and -7.3 kcal/mol for JBU. Specifically, engeletin formed hydrogen bonds with key amino acid residues in the mobile flap region of HPU, including ARG 338, HIS 322, VAL 320, and ASN 168. Similarly, engeletin interacted with the amino acid residues HIS 593, MET 588, MET 637, and GLN 635 of JBU through hydrogen bonding, further highlighting its binding potential.

## 4 Discussion

As a traditional Chinese medicine, SGR is recognized for its properties in detoxification and moisture removal, as well as its ability to dispel wind and enhance joint strength (Fu et al., 2022). The primary constituents encompass flavonoids, phenolic, organic acids, polysaccharides, and other related compounds (Wang et al., 2019a). Notably, astilbin, a type of flavonoids, has significant anti-inflammatory (Fang et al., 2024), anti-gastric cancer (Zhang et al., 2024a), anti-bacterial (Moulari et al., 2006) and analgesic effects (Ilyas et al., 2024). Modern pharmacological studies revealed that the clinical applications of SGR include the treatment of rheumatoid arthritis (Wang et al., 2019b), hepatitis (Hua et al., 2018), urinary tract infections (Huang et al., 2019) and bacterial infections. Moreover, SGR has apparent anti-*H. pylori* and can significantly inhibit the formation of gastric ulcer (Abaidullah et al., 2023), which is consistent with the previously reported protective effect of SGR on gastric mucosal injury. In the present study, our findings revealed that SGR exhibited significant growth-inhibitory activity against the three standard *H. pylori* strains: ATCC 43504, NCTC 26695, and ICDC 111001. This further underscore SGR’s potential as an effective antimicrobial agent against *H. pylori*. Nevertheless, the precise mechanism of SGR against *H. pylori* remains to be clarified.

Ammonia produced through urease hydrolysis modifies the gastric environment and neutralizes gastric acid, thereby facilitating the growth and colonization of *H. pylori* in the stomach (Güzel-Akdemir and Akdemir, 2025), resulting in host damage. Therefore, urease produced by *H. pylori* exerts a crucial function in the pathogenesis of gastric and duodenal ulcers. Currently, the screening of urease depressors for *H. pylori* infection derived from natural Chinese herbs has become as a prominent research topic globally (Aliyeva-Schnorr et al., 2023; Zhang et al., 2024b). Besides, urease is sourced from bacteria, fungi, algae (Righetto et al., 2020), exhibiting monomer structure with varying subunit compositions. Although the sources of urease are different, they have similar amino acid sequence and active site structure. Thus, they share a common catalytic mechanism, characterized by the presence of Ni^2+^ and thiols groups at the active site of urease (Proshlyakov et al., 2021). In this study, JBU was utilized as a model system due to its well-characterized hexametric structure and conserved catalytic mechanism with HPU (over 50% sequence identity in flap regions) (Follmer, 2008; Li et al., 2018). The present proofs testified that SGR could observably inhibit the activities of HPU and JBU in a dose-reliant pattern, suggesting that the enzyme inhibitory activity of SGR is closely associated with its anti-*H. pylori* activity while demonstrating broad-spectrum urease inhibition capability.

JBU exists as a hexamer, with each subunit (91kDa) comprising two Ni^2+^ and fifteen cysteine residues (Mazzei et al., 2021a). The difference is that HPU contains only two types of subunits α (68–73 kDa) and β (8–17 kDa). Moreover, the subunit structure of HPU is characterized by a large, internally hollow quadruplet ((αβ)_3_)_4_) (Kusters et al., 2006). Notably, both enzymes share conserved nickel-containing active sites and essential cysteine residues for flap mobility (Li et al., 2018; Lu et al., 2020), justifying JBU’s use as a pharmacological proxy. In the present study, enzyme kinetics analysis showed that SGR was a non-competitive depressor for HPU and a mixed depressor for JBU, suggesting that the difference in kinetic mechanism may be connected with the structural differences between HPU and JBU. Nevertheless, these differences require much deeper investigation and analysis.

The key to catalyze urease activity lies in its active center nickel ions (Ni^2+^) (Nim et al., 2023) and sulfhydryl (-SH) group (Kumar and Kayastha, 2010). In this study, two types of protectors were employed, one being thiol compounds and the other being inorganic compounds which affect urease activity by different mechanisms. Generally, thiols reagent, including DTT, GSH, and L-cys, interacts with the sulfhydryl groups located at the active site. On the other hand, inorganic compounds such as NaF and BA interact with Ni^2+^ to inhibit the binding of the depressor to the active site of urease. The combination of SH-blocking reagents or competitive Ni²^+^ compounds with the depressor has been widely used to investigate the potential urease inhibition targets of depressors (Lu et al., 2020). For instance, He et al. (2022) demonstrated that coptisine can interact with Ni²^+^, the active center of urease, as well as with the essential sulfhydryl group within the active site, thereby inhibiting urease activity. Lu et al. (2022) demonstrated that sanguinarine significantly inhibits HPU activity by targeting sulfhydryl and Ni^2+^. Yu et al. (2015) reported that patchouli alcohol inhibited urease activity through interactions with sulfhydryl groups. The findings of this study indicated that sulfhydryl reagents including DTT and GSH exhibited effective protective roles against HPU and JBU. In addition, compared with SGR-induced enzyme activity, BA and NaF have synergistic inhibitory effects on HPU and JBU. These results suggest that the repressive mechanism of SGR towards HPU and JBU may be related to the blocking of sulfhydryl active sites. Nevertheless, further investigation is required to probe the mechanisms underlying the inhibitory roles of SGR on urease.

For further proving whether the urease inhibition by SGR is reversible, GSH was used for the reactivation test. Results of the curve analysis confirmed that both enzymatic activities were reversible. Specifically, SGR-blocked HPU and JBU activity could be reactivated by GSH. Notably, both HPU and JBU activities demonstrated recovery to 40% of their initial levels. Restoration of SGR-modified urease activity by sulfhydryl compounds further supports the crucial role of sulfhydryl groups at the active site in SGR-induced urease inhibition. The results were consistent with those reported in previous studies (Lu et al., 2022; Tan et al., 2017).

Flavonoids were the major chemical components of SGR. In particular, flavonoids such as astilbin, neoastilbin, isoastilbin, neoisoastilbin, engeletin, and isoengeletin demonstrated significant anti-inflammatory (Zhan et al., 2024) and anti-bacterial properties (Sharma et al., 2020). The results of enzyme activity inhibition showed that astilbin and engeletin exhibited a certain inhibitory effect on urease activity. Consistent with urease inhibition by SGR, astilbin exhibited a non-competitive depressor to HPU, and a mixed depressor to JBU. Notably, this kinetic congruence between the crude extract and its purified marker compound (astilbin) suggests that the observed inhibition patterns in SGR primarily reflect the combined effects of its key flavonoid constituents, though the apparent kinetic parameters represent composite values from all bioactive components. In addition, molecular docking simulation technology offers enhanced technical support for validating the rationale behind the underlying mechanisms. The results indicated that astilbin and engeletin form tight hydrogen bonds and hydrophobic contacts with several amino acid residues located on the mobile flap of HPU, fixing the helix-turn-helix motif atop the active site pocket. This results in the stabilization of the flap conformation in an open state and ultimately in the inactivation of the enzyme. The docking results further support that astilbin’s specific binding mode can dominate the overall inhibition kinetics even in the complex SGR matrix, as evidenced by the consistent non-competitive patterns between purified astilbin and the whole extract. Research results indicated that astilbin and engeletin are closely associated with the anti-urease activity of SGR. While these findings highlight the major contributors to SGR’s urease inhibition, we acknowledge that minor constituents may influence the overall activity. This reflects a characteristic pharmacological feature of herbal extracts, which warrants further systems-level investigation in the future.

## 5 Conclusion

This study demonstrated significant growth inhibition of *H. pylori* by SGR. Moreover, our experimental findings indicated that SGR exerted a significant inhibitory role towards HPU and JBU in a concentration-reliant pattern. Flavonoids including astilbin and engeletin are the main active ingredients of SGR-induced urease inactivation. Enzyme kinetic analysis showed that SGR was a slow binding, non-competitive suppressant to HPU, and a slow binding, mixed suppressant to JBU. In-depth mechanistic studies uncovered that sulfhydryl groups at the active site of urease are responsible for the enzyme inactivation by SGR. SGR has shown significant potential in the medical field the remedy of gastroenteric diseases associated with *H. pylori* infection. In this experiment, we provide effective scientific evidence support for the traditional Chinese medicine SGR in the *H. pylori*-associated gastrointestinal diseases.

## Data Availability

The original contributions presented in the study are included in the article/supplementary material. Further inquiries can be directed to the corresponding authors.
